# The Influence of the 1-(3-Trifluoromethyl-Benzyl)-1*H*-Pyrazole-4-yl Moiety on the Adenosine Receptors Affinity Profile of Pyrazolo[4,3-*e*][1,2,4]Triazolo[1,5-*c*]Pyrimidine Derivatives

**DOI:** 10.1371/journal.pone.0143504

**Published:** 2015-12-01

**Authors:** Stephanie Federico, Sara Redenti, Mattia Sturlese, Antonella Ciancetta, Sonja Kachler, Karl-Norbert Klotz, Barbara Cacciari, Stefano Moro, Giampiero Spalluto

**Affiliations:** 1 Dipartimento di Scienze Chimiche e Farmaceutiche, Università degli Studi di Trieste, Trieste, Italy; 2 Molecular Modeling Section (MMS), Dipartimento di Scienze del Farmaco, Università degli Studi di Padova, Padova, Italy; 3 Institut für Pharmakologie und Toxicologie, Universität Würzburg, Würzburg, Germany; 4 Dipartimento di Scienze Chimiche e Farmaceutiche, Università degli Studi di Ferrara, Ferrara, Italy; University of North Dakota, UNITED STATES

## Abstract

A new series of pyrazolo[4,3-*e*][1,2,4]triazolo[1,5-*c*]pyrimidine (PTP) derivatives has been developed in order to explore their affinity and selectivity profile at the four adenosine receptor subtypes. In particular, the PTP scaffold was conjugated at the C2 position with the 1-(3-trifluoromethyl-benzyl)-1*H*-pyrazole, a group believed to confer potency and selectivity toward the human (h) A_2B_ adenosine receptor (AR) to the xanthine ligand 8-(1-(3-(trifluoromethyl)benzyl)-1*H*-pyrazol-4-yl)-1,3-dimethyl-1*H*-purine-2,6(3*H*,7*H*)-dione (CVT 6975). Interestingly, the synthesized compounds turned out to be inactive at the hA_2B_ AR but they displayed affinity at the hA_3_ AR in the nanomolar range. The best compound of the series (**6**) shows both high affinity (hA_3_ AR K_i_ = 11 nM) and selectivity (A_1_/A_3_ and A_2A_/A_3_ > 9090; A_2B_/A_3_ > 909) at the hA_3_ AR. To better rationalize these results, a molecular docking study on the four AR subtypes was performed for all the synthesized compounds. In addition, CTV 6975 and two close analogues have been subjected to the same molecular docking protocol to investigate the role of the 1-(3-trifluoromethyl-benzyl)-1*H*-pyrazole on the binding at the four ARs.

## Introduction

**Fig 1:**
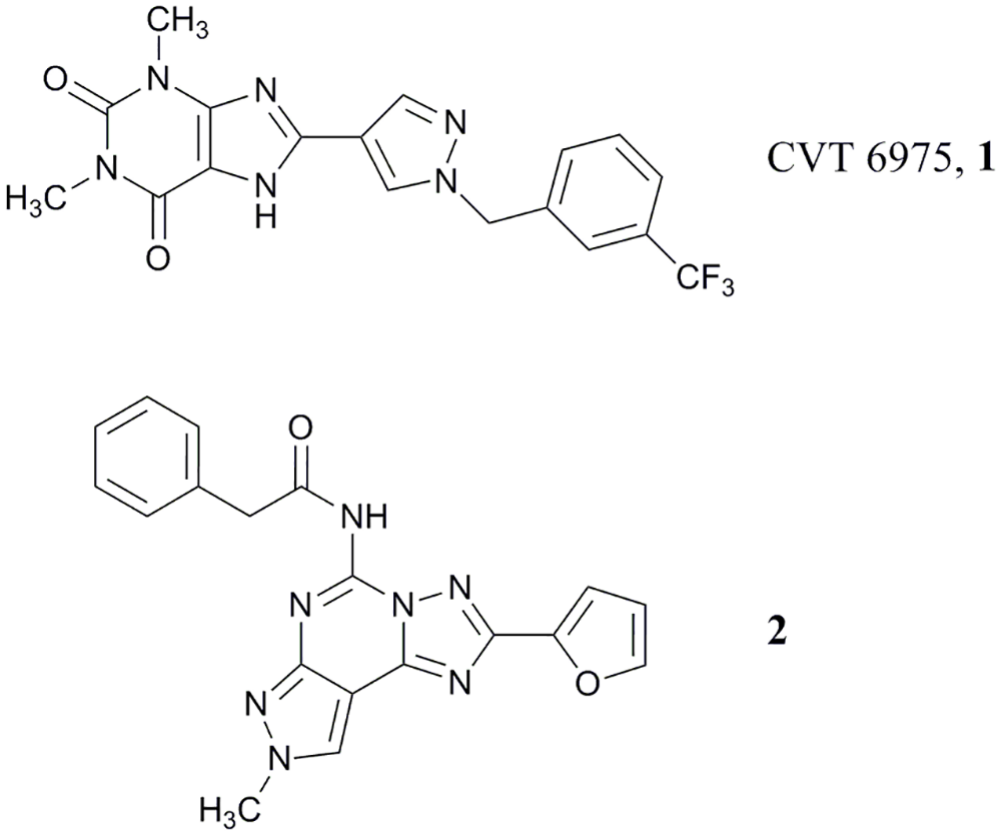
CVT6975 (1) and the pyrazolo[4,3-*e*][1,2,4]triazolo[1,5-*c*]pyrimidine derivative 2 as potent A_2B_ and A_3_ AR antagonists, respectively.

Adenosine is an ubiquitous modulator, which exerts its functions through interaction with four G protein-coupled receptors classified as A_1_, A_2A_, A_2B_ and A_3_ adenosine receptors (ARs). [1,2] While A_1_, A_2A_ and A_3_ ARs are stimulated by low concentrations of adenosine, the A_2B_ subtype requires higher concentrations of the natural ligand. [3] In recent years, numerous potent and selective ligands (agonists and antagonists) for the A_1_, A_2A_, A_2B_ and A_3_ AR subtypes have been discovered. [4,5] Some of them served as pharmacological tools and demonstrated potential therapeutic applications in various pathological processes. For example, human (h) A_1_ AR agonists are useful against cardiac arrhythmias and pain, while antagonists are beneficial in kidney failure. Antagonists for the hA_2A_ AR were widely studied for the treatment of Parkinson’s disease and other neurodegenerative disorders. [3–5] To date, the physio-pathological role of the hA_3_ AR is controversial. This AR subtype is involved in cancer and inflammation, although it is not yet clear whether blocking or activating this receptor would be therapeutic for these conditions. [6] Identification of potent and selective ligands for the hA_2B_ AR is difficult as compared with the other receptor subtypes. In addition, its characterization as a low-affinity adenosine receptor led to the erroneous belief that the A_2B_ AR was not of substantial physiological relevance. [7–9] As a consequence, the hA_2B_ AR is the least characterized among the ARs, although in the last years, significant advances have been made in the understanding of the molecular pharmacology and physiology of the hA_2B_ ARs. [7–9] This subtype is highly expressed during inflammatory and hypoxic conditions and, in these circumstances, adenosine reaches very high extracellular concentrations necessary to activate the hA_2B_ AR subtype. [9] In fact, the hA_2B_ ARs seem to be implicated in many different patho-physiological conditions such as: asthma, [10–14] intestinal functions, vascular tone and permeability, ischemic preconditioning, inflammation, angiogenesis and sickle disease. [9,15–20] Therefore, hA_2B_ AR antagonists have been proposed as anti-asthmatic and/or anti-inflammatory agents. [4,5] In particular, most of the so far developed hA_2B_ AR antagonists are xanthine derivatives or analogues. [21] In 2006 a very interesting compound named CVT 6975 (8-(1-(3-(trifluoromethyl)benzyl)-1*H*-pyrazol-4-yl)-1,3-dimethyl-1*H*-purine-2,6(3*H*,7*H*)-dione (**1**, Fig 1) proved to be one of the most potent A_2B_ AR antagonists ever reported. [22] In the non-xanthine family, some adenine [23], 7-deazapurines [24], triazolo-triazine, [24] quinazoline, [25], triazinobenzimidazole [26], pyrimidine [27] and pyrazine [28] derivatives have been reported as hA_2B_ AR antagonists, but few of the synthesized compounds (e.g. pyrimidines) showed both significant potency and selectivity for this receptor subtype.

**Fig 2:**
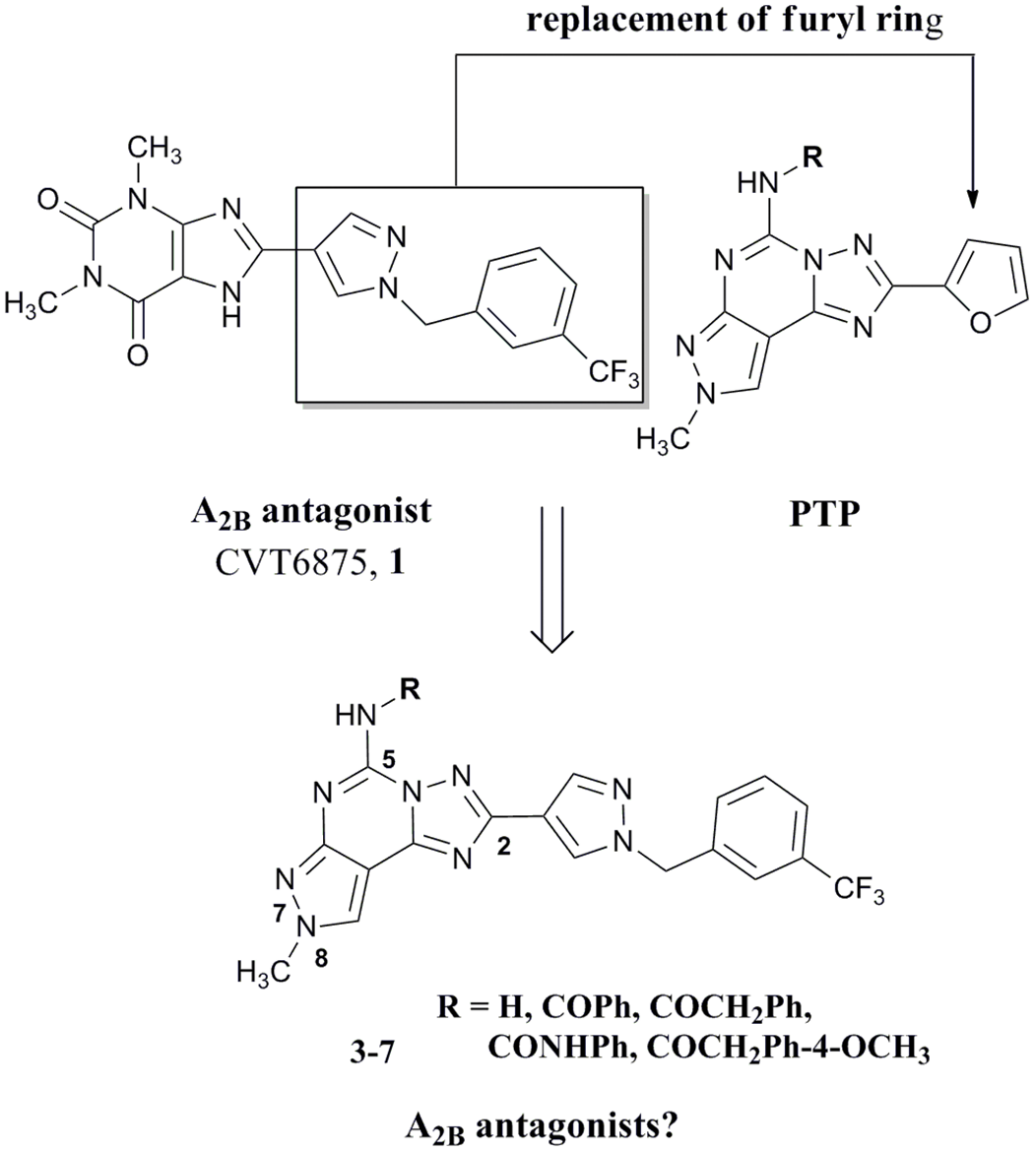
Rational for the design of the target compounds 3–7.

In one of our research programs aimed at the identification of new non-xanthine hA_2B_ AR antagonists, we investigated variously substituted pyrazolo[4,3-*e*][1,2,4]triazolo[1,5-*c*]pyrimidine (PTP) derivatives. [29–31] A large number of derivatives with different substitutions at the N7, N8 and N5 positions (see Fig 2 for numbering of the PTP ring system) have been prepared, but unfortunately none of the synthesized compounds showed both affinity and selectivity at the hA_2B_ ARs. [29–31] One of the most interesting compounds developed is derivative **2** (Fig 1), which displays good affinity for the hA_2B_ AR (K_i_ = 165 nM) but it shows a greater affinity towards the hA_3_ AR (K_i_ = 0.81 nM). [32] Taking into account these experimental observations, we decided to synthesize new derivatives by replacing the furan moiety in position C2 of the PTP scaffold with the 1-(3-trifluoromethyl-benzyl)-1*H*-pyrazole-4-yl moiety present in the CVT 6975 (**1**) with the aim to better explore the role of this position in the receptor recognition and possibly obtain new A_2B_ AR antagonists with non xanthinic structure (Fig 2).

## Materials and Methods

### Chemistry

#### General

Reactions were routinely monitored by thin-layer chromatography (TLC) on silica gel (precoated F254 Merck plates). Flash chromatography was performed using Merck 60–200 mesh silica gel. Infrared spectra (IR) were measured on a Perkin Elmer 257 instrument. ^1^H-NMR were determined in CDCl_3_ or DMSO-d_6_ solutions with a Varian Gemini 200 spectrometer, peaks positions are given in parts per million (δ) downfield relative to the central peak of the solvent; J values are given in Hz. Melting points were determined on a Buchi-Tottoli instrument and are uncorrected. All reported products showed IR and ^1^H NMR spectra in agreement with the assigned structures. Electrospray mass spectra were recorded on a ESI Bruker 4000 Esquire spectrometer and compounds were dissolved in methanol. Elemental analyses were performed by the microanalytical laboratory of Dipartimento di Scienze Chimiche e Farmaceutiche, University of Trieste, and they were within ±0.4% of the theoretical values for C, H and N. Light petroleum ether refers to the fractions boiling at 40–60°C. Organic solutions were dried over anhydrous magnesium sulfate.

#### Synthesis of 1-(3-trifluoromethyl-benzyl)-1*H*-pyrazole-4-carboxylic acid hydrazide (10)

Pyrazole ester derivative **9** (5.7 g, 20 mmol) was dissolved in absolute ethanol (30 mL) and an equivalent amount of hydrazine monohydrate (0.96 mL, 20 mmol) was added and the resulting mixture was stirred at reflux for three days. The solvent was then removed in vacuo and the residue crystallized from EtOAc to afford the corresponding hydrazide **9** as a white solid (mp 95°C) in quantitative yield. IR (KBr): 3445–2960, 1690, 1650, 1610, 1480 cm^-1^; ^1^H NMR (CDCl_3_) δ: 3.65 (bs, 2H); 5.21 (s, 2H); 6.51 (bs, 1H); 7.21–7.58 (m, 4H); 7.66 (s, 1H); 7.77 (s, 1H). Anal. Calcd. for C_12_H_11_N_4_OF_3_ (MW 284.24): C, 50.71; H, 3.90; N, 19.17. Found: C, 50.53; H, 3.85; N, 19.18.

#### Synthesis of 8-methyl-2-[1-(3-trifluoromethyl-benzyl)-1*H*-pyrazol-4-yl]-8*H*-pyrazolo[4,3-*e*][1,2,4]triazolo[1,5-*c*]pyrimidin-5-ylamine (3)

Pyrazole **8** (2.4 g, 20 mmol) was dissolved in diphenyl ether (30 mL) and hydrazide **10** (6.25 g, 22 mmol, 1.1 eq) was added. The mixture was heated at 260°C using a Dean-Stark for the azeotropic elimination of water produced in the reaction. After 2.5 h, the mixture was poured onto hexane (200 mL) and cooled. The precipitate of crude pyrazole-triazole derivative **11** was filtered off, and utilized for the next step without further purifications. The crude residue, was dissolved in N-methyl pyrrolidone (40 mL), cyanamide (3 eq, 60 mmol, 2.5 g) and *p*-toluen sulfonic acid (1.5 eq, 30 mmol, 5.7 g) were added and the mixture was heated at 160°C for 4 h. Cyanamide (3 eq, 60 mmol, 2.5 g) was added again and the solution was heated overnight. Then the solution was diluted with EtOAc (80 mL) and the precipitate (excess of cyanamide) was filtered off; the filtrate was concentrated under reduced pressure and washed with water (3 x 30 mL). The organic layer was dried (Na_2_SO_4_) and evaporated under vacuum. The residue was purified by flash chromatography (EtOAc/Methanol 9.5:0.5) to afford the desired compound **3** in a good overall yield (60%) as a yellow solid. Mp 125°C (EtOAc-light petroleum); IR (KBr): 3330–2960, 1640, 1605, 1550, 1450 cm^-1^; ^1^H NMR (DMSO-d_6_) δ: 3.87 (s, 3H); 5.20 (s, 2H); 5.72 (bs, 2H); 7.18–7.41 (m, 4H); 7.84 (s, 1H); 7.96 (s, 1H); 7.97 (s, 1H). ES-MS: (MH^+^) 414.4. Anal. Calcd. for C_18_H_14_N_9_F_3_ (MW 413.36): C, 52.30; H, 3.41; N, 30.50. Found: C, 52.13; H, 3.44; N, 30.38.

#### Synthesis of 1-{8-Methyl-2-[1-(3-trifluoromethyl-benzyl)-1*H*-pyrazol-4-yl]-8*H*-pyrazolo[4,3-*e*][1,2,4]triazolo [1,5-*c*]pyrimidin-5-yl}-3-phenylurea (4)

Amino compound (**3**) (206 mg, 0.5 mmol) was dissolved in freshly distilled dioxane (15 mL) and phenyl isocyanate (130 μL, 1 mmol, 2 eq) was added. The mixture was refluxed under argon for 18 hours. Then the solvent was removed under reduced pressure and the residue was purified by flash chromatography (EtOAc-light petroleum 8:2) to afford the desired compound **4** in a good yield (88%) as a white solid, mp 180°C (EtOAc-light petroleum); IR (KBr): 3355–2980, 1705, 1655, 1600, 1530 cm^-1^; ^1^H NMR (CDCl_3_) δ: 3.97 (s, 3H); 5.22 (s, 2H); 7.01–7.42 (m, 9H); 7.88 (s, 1H); 7.98 (s, 1H); 7.99 (s, 1H); 8.30 (bs, 1H); 10.93 (bs, 1H). ES-MS: (MH^+^) 533.1. Anal. Calcd. for C_25_H_19_N_10_OF_3_ (MW 532.48): C, 56.39; H, 3.60; N, 26.30. Found: C, 56.53; H, 3.62; N, 26.48.

#### General procedure for acylation of amino group at the 5 position (5–7)

Amino compound **3** (206 mg, 0.5 mmol) was dissolved in freshly distilled dioxane (15 ml) and the appropriate acyl chloride (1 mmol) and triethylamine (1 mmol, 140 μL) were added. The mixture was refluxed under argon for 18 hours. Then the solvent was removed under reduced pressure and the residue was dissolved in EtOAc (30 ml) and washed twice with water (15 ml). The organic phase was dried on anhydrous Na_2_SO_4_ and concentrated under reduced pressure. The residue was purified by flash chromatography (EtOAc-light petroleum 8:2) to afford the desired compounds (**5–7**).

*N-{8-Methyl-2-[1-(3-trifluoromethyl-benzyl)-1H-pyrazol-4-yl]-8H-pyrazolo[4*,*3-e][1*,*2*,*4]triazolo[1*,*5-c]pyrimidin-5-yl}-benzamide* (**5**): Yield 85%, pale yellow solid; mp 115°C (EtOAc-light petroleum); IR (KBr): 3240–2995, 1685, 1635, 1570, 1525 cm-1; ^1^H NMR (CDCl_3_) δ: 3.96 (s, 3H); 5.22 (s, 2H); 7.08–7.55 (m, 9H); 7.85 (s, 1H); 7.94 (s, 1H); 7.97 (s, 1H); 9.41 (bs, 1H). ES-MS: (MH^+^) 518.2. Anal. Calcd. for C_25_H_18_N_9_OF_3_ (MW 517.47): C, 58.03; H, 3.51; N, 24.36. Found: C, 57.92; H, 3.44; N, 24.18.

*N-{8-Methyl-2-[1-(3-trifluoromethyl-benzyl)-1H-pyrazol-4-yl]-8H-pyrazolo[4*,*3-e][1*,*2*,*4]triazolo[1*,*5-c]pyrimidin-5-yl}-phenyl-acetamide* (**6**): Yield 85%, white solid; mp 190°C (EtOAc-light petroleum); IR (KBr): 3240–2990, 1675, 1605, 1580, 1525 cm-1; ^1^H NMR (CDCl_3_) δ: 3.96 (s, 3H); 4.27 (s, 2H); 5.20 (s, 2H); 7.03–7.37 (m, 9H); 7.82 (s, 1H); 7.94 (s, 1H); 7.97 (s, 1H); 8.82 (bs, 1H). ES-MS: (MH^+^) 518.2. Anal. Calcd. for C_26_H_20_N_9_OF_3_ (MW 531.49): C, 58.76; H, 3.79; N, 23.72. Found: C, 58.93; H, 3.65; N, 23.88.

*2-(4-Methoxy-phenyl)-N-{8-Methyl-2-[1-(3-trifluoromethyl-benzyl)-1H-pyrazol-4-yl]-8H-pyrazolo[4*,*3-e] [1*,*2*,*4]triazolo[1*,*5-c]pyrimidin-5-yl}-phenyl-acetamide* (**7**): Yield 77%, yellow solid; mp 178°C (EtOAc-light petroleum); IR (KBr): 3245–2975, 1680, 1615, 1570, 1515 cm-1; ^1^H NMR (CDCl_3_) δ: 3.97 (s, 3H); 4.18 (s, 3H); 4.25 (s, 2H); 5.21 (s, 2H); 7.03–7.37 (m, 6H); 7.57 (d, 2H, J = 9); 7.83 (s, 1H); 7.96 (s, 1H); 7.98 (s, 1H); 8.78 (bs, 1H). ES-MS: (MH^+^) 562.2. Anal. Calcd. for C_27_H_22_N_9_O_2_F_3_ (MW 561.52): C, 57.75; H, 3.95; N, 22.45. Found: C, 57.53; H, 3.86; N, 22.33.

### Biology

All pharmacological methods followed the procedures as described earlier. [33–36] In brief, membranes for radioligand binding were prepared from CHO cells stably transfected with human AR subtypes in a two-step procedure. In a first low-speed step (1,000 x g) cell fragments and nuclei were removed. The crude membrane fraction was sedimented from the supernatant at 100,000 x g. The membrane pellet was resuspended in the buffer used for the respective binding experiments (50 mM Tris/HCl buffer pH 7.4 for hA_1_ and hA_2A_ AR; 50 mM Tris/HCl, 10 mM MgCl_2_, 1 mM EDTA, pH 8.25 for hA_3_ AR), frozen in liquid nitrogen and stored at -80°C. For the measurement of adenylyl cyclase activity only one high speed centrifugation of the homogenate was used. The resulting crude membrane pellet was resuspended in 50 mM Tris/HCl, pH 7.4 and immediately used for the cyclase assay.

For radioligand binding at human A_1_ ARs 1 nM [^3^H]CCPA was used (K_D_ = 0.61 nM), whereas 10 nM [^3^H]NECA were used for A_2A_ (K_D_ = 20.1 nM). The high-affinity agonist [^3^H]HEMADO (K_D_ = 1.1 nM) was used for A_3_ AR binding at a concentration of 1 nM. Non specific binding of [^3^H]CCPA was determined in the presence of 1 mM theophylline, in the case of [^3^H]NECA and [^3^H]HEMADO, 100 μM R-PIA was used. K_i_-values from competition experiments were calculated with the program SCTFIT. [33–35]

Radioligand binding at human A_2B_ ARs is problematic as no high-affinity radioligand is commercially available for this subtype. Therefore, inhibition of NECA-stimulated adenylyl cyclase activity (stimulation with 5 μM of NECA, EC_50_ = 2.4 μM) was determined as a measurement of affinity of compounds. The procedure was carried out as described previously with minor modifications. [33–35] Membranes were incubated with about 150,000 cpm of [α-^32^P]ATP for 20 min in the incubation mixture as described without EDTA and NaCl. [33–35]

### Molecular modeling

The models for human A_1_, A_2A_, A_2B_ and A_3_ ARs were retrieved from the Adenosiland platform (http://mms.dsfarm.unipd.it/Adenosiland) [36], selecting the crystallographic structure of hA_2A_ AR, in complex with the high affinity antagonist ZM241385 (PDB access code: 4EIY; resolution 1.8 Å), as template for homology modelling. [37] The numbering of the amino acids follows the arbitrary scheme by Ballesteros and Weinstein: each amino acid identifier starts with the helix number, followed by the position relative to a reference residue among the most conserved amino acids in that helix, to which the number 50 is arbitrarily assigned. [38]

Ligand conformations were built using the MOE-builder tool implemented in the MOE suite [39] and were subjected to a MMFF94x energy minimization until the rmsd conjugate gradient was <0.05 kcal mol^–1^ Å^–1^. All antagonists were docked into the hypothetical TM binding site of the hA_3_ AR model derived from the centre of mass of the ZM241385 inhibitor. The workflow of the molecular docking approach used in the present work has been previously reported. [40] Molecular docking study was performed employing the docking tool of the GOLD Suite v5.2.1. [41] For each compound, 25 independent docking runs were performed and searching was conducted within a user-specified docking sphere with the Genetic Algorithm protocol and the GoldScore scoring function. The resulting docking complexes (ligand and side-chain residues within 4.5 Å) were subjected to a MMFF94x energy minimization until the rms conjugate gradient was <1 kcal mol^–1^ Å^–1^.

Analyses of docking poses and quantitative analysis for nonbonded intermolecular interactions (H-bonds, hydrophobic, electrostatic) were calculated and visualized using MOE suite. [39] To estimate the electrostatic contributions, atomic charges for the ligands were calculated with MOPAC-2012 [42] and the PM3/ESP methodology, whereas partial charges for the protein amino acids were computed with the AMBER99 force field. The validation of the proposed docking protocol has been previously published. [43] Physicochemical and ADME properties of references and newly synthesized compounds were calculated with StarDropTM, version 6.0. [44]

## Results and Discussion

### Chemistry

**Fig 3:**
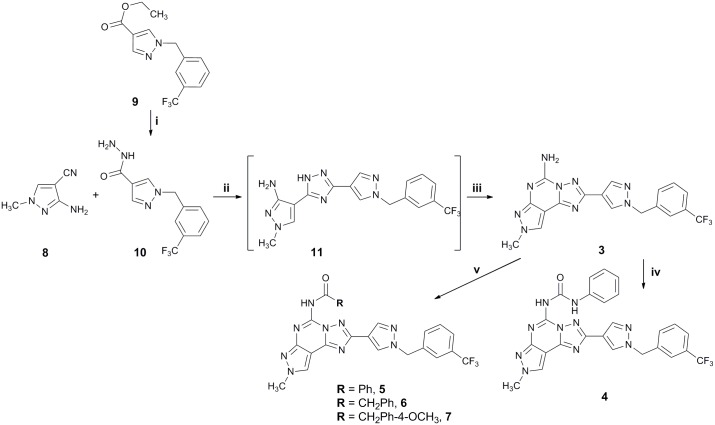
Synthesis of desired compounds 3–7. *Reagent*s: **i**: NH_2_NH_2_^.^H_2_O, EtOH, reflux, 3 days; **ii**: Ph_2_O, 260°C, 2.5 h; **iii**: NH_2_CN, pTsOH, 160°C, 4 h; **iv**: PhNCO, dioxane, reflux; **v**: RCOCl, Et_3_N, dioxane, reflux.

Compounds **3–7** were prepared following the general synthetic strategy summarized in Fig 3. They were synthesized according to a well-known procedure for the synthesis of the PTPs, previously reported. [45–47]

Reaction of the *N* ^*2*^-methyl-4-cyano-5-amino pyrazole **8** with the hydrazide derivative **10** (prepared from the corresponding ester **9** [22] by reaction with hydrazine monohydrate in absolute ethanol at reflux for three days) in diphenyl ether at 260°C led to the aminotriazole **11**, which was in turn converted, without any further purification, into the final compound **3** by reaction with cyanamide in the presence of *p*-toluen sulfonic acid. Amido derivatives **5–7** were obtained by reaction of the amino compound **3** with the appropriate acyl halide in dioxane at reflux in the presence of triethylamine, while the ureido derivative **4** was obtained by reaction of the amino compound **3** with phenyl isocyanate in dioxane at reflux (Fig 3).

### Biological activity

**Fig 4:**
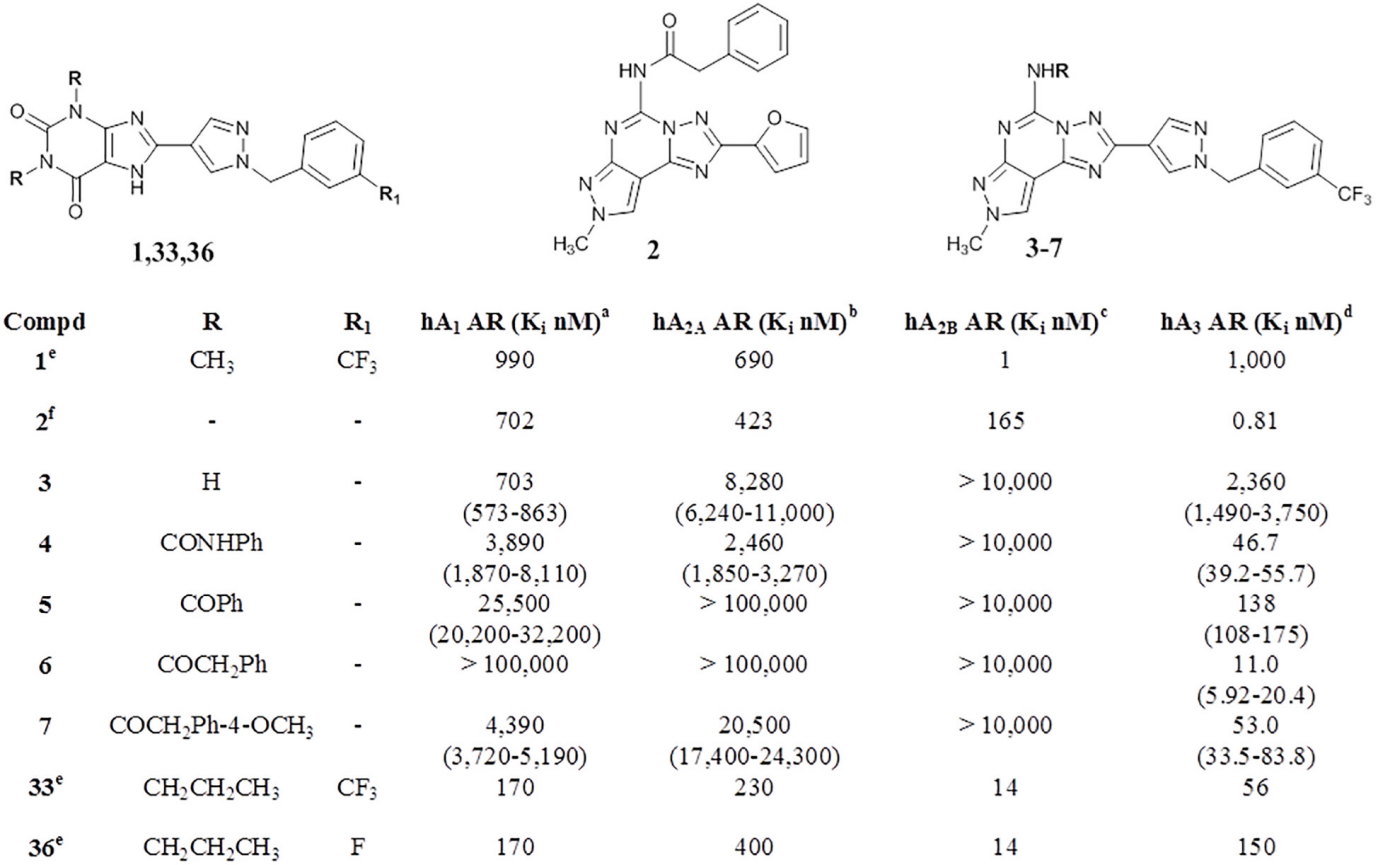
Structures and binding profile of reference (1,2,33,36) and synthesized compounds (3–7). ^a^Displacement of specific [^3^H]-CCPA binding at hA_1_ ARs expressed in CHO cells, (n = 3–6). ^b^Displacement of specific [^3^H]-NECA binding at hA_2A_ ARs expressed in CHO cells. ^c^K_i_ values of the inhibition of NECA-stimulated adenylyl cyclase activity in CHO cells expressing hA_2B_ ARs. ^d^Displacement of specific [^3^H]-HEMADO binding at hA_3_ ARs expressed in CHO cells. Data are expressed as geometric means, with 95% confidence limits. ^e^data from ref. [22]. ^f^data from ref. [32].

Newly synthesized compounds (**3–7**) were tested at the human A_1_, A_2A,_ A_2B_ and A_3_ ARs expressed in CHO (Chinese Hamster Ovary) cells. [^3^H]CCPA ([^3^H]2-Chloro-*N* ^*6*^-cyclopentyladenosine) (hA_1_ AR), [^3^H]NECA ([^3^H]5'-*N*-ethylcarboxamidoadenosine) (hA_2A_ AR) and [^3^H]HEMADO ([^3^H]2-(1-Hexynyl)-*N*-methyladenosine) (hA_3_ ARs) were used as radioligands in binding assays. Inhibition of NECA-stimulated adenylyl cyclase activity in CHO cells expressing hA_2B_ receptors was determined as a measurement of affinity of compounds at the hA_2B_ AR (Fig 4). [33–35]

As clearly depicted in Fig 4, surprisingly all novel CVT-like compounds do not show appreciable activity at the hA_2B_ AR. On the contrary they have shown an unexpected high affinity, with some in the nanomolar range, towards the hA_3_ AR subtype. In S1 Fig representative competition curves for the hA_3_ selective agonist [^3^H]HEMADO (total binding) from single experiments for compounds **6** and **4**, respectively, are reported. The affinity at the hA_3_ AR seems to be more related to the substitution at the N5 position than to the substituent present at the C2 position. In fact, the N5-unsubstituted derivative **3**, proved to be almost inactive at all four ARs, while, the introduction of a substituent at the N5 position (**4**,**7**) induced a recovery of affinity at the hA_3_ AR with good levels of selectivity versus the other receptor subtypes. In particular, the introduction of an arylcarbamoyl moiety, which has been demonstrated optimal substituent for the hA_3_ AR, [29–32,45–48] led to compound **4**, which shows a K_i_ value of 46.7 nM with a 50–70 fold selectivity versus the other receptor subtypes. The most potent compound of this series (**6**) is the derivative bearing a phenylacetyl moiety at the N5 position which shows a K_i_ value of 11.1 nM and high selectivity (hA_1_/hA_3_ and hA_2A_/hA_3_ >9090; hA_2B_/hA_3_ >909) over the other receptor subtypes. Introduction of a methoxy group on the *para* position of phenyl ring (**7**) leads to a reduction of affinity of about 5 fold (hA_3_ AR K_i_ = 53.0 nM) with a consequent reduction of selectivity, while the replacement of phenylacetyl group with a benzoyl moiety (**5**) considerably reduces both affinity and selectivity at the hA_3_ AR.

Regarding the C2 position, in a previous work it has been demonstrated that the substitution of the furyl ring at the C2 position of the PTP scaffold with a phenyl moiety gives derivatives which maintained high affinity at the hA_3_ AR and showed improved selectivity at this AR subtype. [48] Moreover, with this work we have demonstrated that at the hA_3_ AR bigger substituents than five or six-membered aromatic rings, such as the long chain of 1-benzyl-1*H*-pyrazole, are also tolerated. Obviously, this short series of compounds does not allow us to outline a structure activity relationship (SAR) analysis. In addition, the obtained results are consistent with the well-known SAR of PTP derivatives at the hA_3_AR, that is: at the N5 position a substitution on the amino group is preferred and, in particular, phenylacetamido moieties are better than benzamido moieties; whereas, at the C2 position, the substitution of the furyl ring enhances selectivity versus the other receptor subtypes. [29–32,45–48] It is worth to remember that although no A_3_ AR antagonist has still reached clinical trials, there are several pharmacological evidences that suggest their potential use for the treatment of asthma, chronic obstructive pulmonary disease (COPD), neurodegenerative diseases, stroke, glaucoma and cancer. [6] Consequently there is an ongoing interest in the development of novel potent and selective antagonists for the A_3_ AR subtype.

### Computational studies

**Fig 5:**
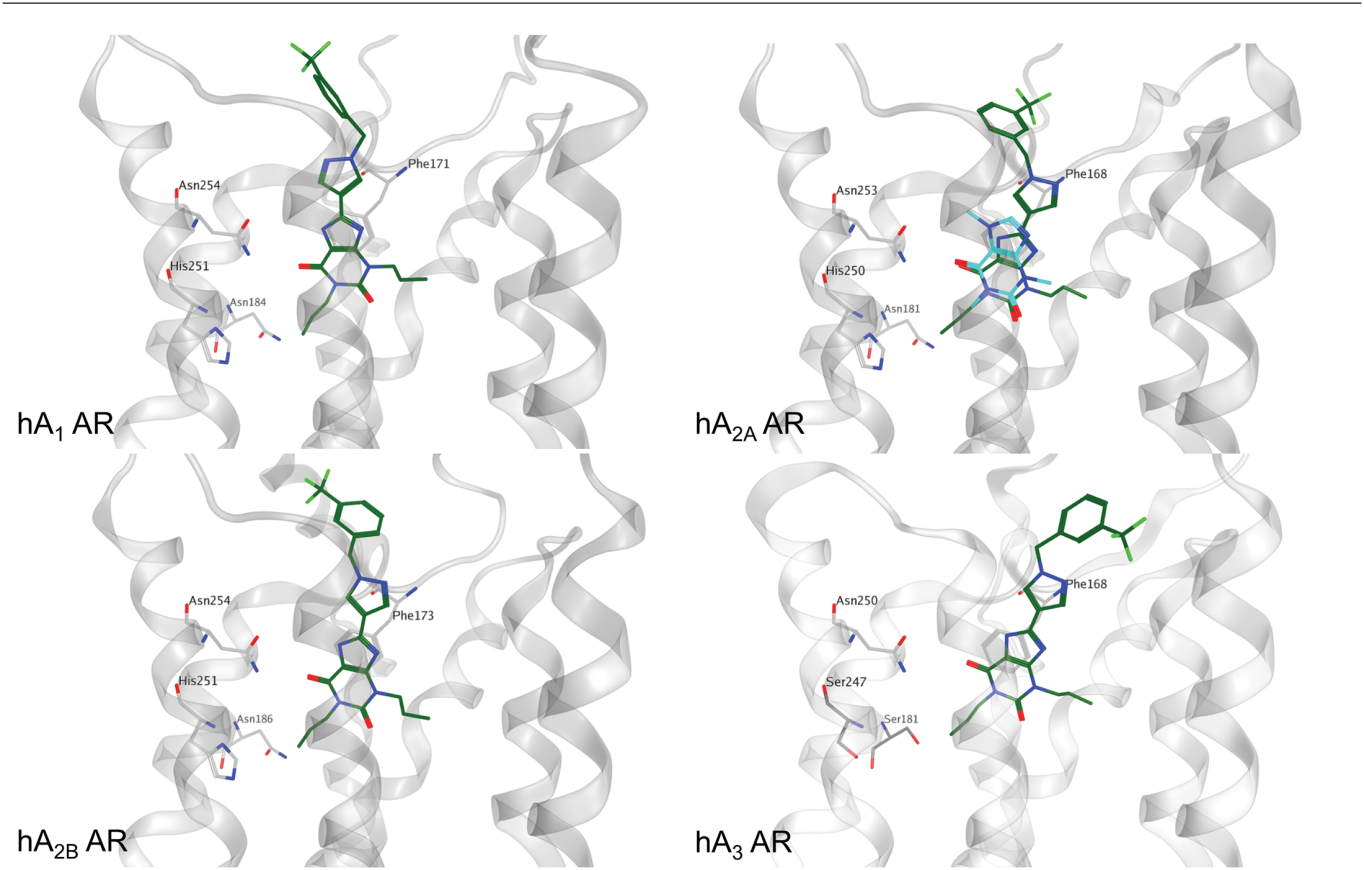
Binding mode of xanthine-based compounds at the four AR subtypes. Compound **36**, in dark green, was selected as reference to show the proposed binding mode at the four AR subtypes. The crystallographic coordinates of caffeine, in magenta, bound to hA_2A_ AR are reported superimposed to the binding mode of compound **36**. The xanthine core of compound **36** is oriented in a similar manner to the crystallographic data. Residues particularly important in the binding are reported as light grey sticks.

To better rationalize these unexpected experimental results, a molecular docking study on the four hAR subtypes was performed. The workflow of the molecular docking approach used in the present work has been previously reported. [40,49] First of all, the role of the 1-(3-trifluoromethyl-benzyl)-1*H*-pyrazole-4-yl moiety in receptor recognition has been investigated starting from the analysis of the hypothetical binding mode of CTV 6975 (**1**) and two other structurally similar analogues **36** and **33** [22] against all four AR subtypes (Fig 5A). In particular, compound **36** differs in the 1,3-disubstitution of the xanthine core, where the methyl groups of CVT 6975 (**1**) are replaced by two propyl groups. Compound **33** shares the same substituents on the xanthine core with **36** with the trifluoromethyl group being replaced by a fluorine atom. Both compounds retain a notable affinity for the hA_2B_ AR (K_i_ = 14 nM) but a lower selectivity towards the other subtypes (**36**: hA_1_ AR K_i_ = 170 nM; hA_2A_ AR K_i_ = 400 nM; hA_3_ AR K_i_ = 150 nM, **33:** hA_1_ AR K_i_ = 170 nM; hA_2A_ AR K_i_ = 230 nM; hA_3_ AR K_i_ = 56 nM). [22]

Molecular docking results suggest for the three CVT analogues (**1**, **36, 33**) an univocal binding mode into the putative orthosteric pocket of the hA_2B_ AR with the xanthine core faced to the key residue Asn254 (6.55) and sided by Phe173 (EL2), Val250 (6.51) and Ile276 (7.39) (Fig 5B). The 1-benzyl-1*H*-pyrazole moiety whether substituted with trifluoromethyl or fluorine atom interacts with Met272 (7.35) and with residues located in EL2, namely Leu172-Glu174. As an example, the hypothetical binding mode of compound **36** at the hA_2B_ AR is reported in Fig 5B. Interestingly, the docking results of compound **36** at the hA_1_, hA_2A_ and hA_3_ ARs confirm a similar recognition pathway to that observed for the A_2B_ AR (Fig 5B). In particular, the xanthine core is positioned closely to that observed for two other xanthinic ligands, caffeine and XAC, in their crystallographic bound state at the hA_2A_ AR (PDB codes 3RFM and 3REY, respectively). [50] Moreover, molecular docking results suggest a crucial role of the 1-benzyl-1*H*-pyrazole moiety in increasing the binding affinity against all the receptor subtypes, while the nature of the 1,3-disubstitution at the xanthine core seems to play a role in determining the ligand selectivity profile. In fact, the presence of larger substituents in 1,3 positions, such as propyl groups, seems to be well tolerated at the hA_1_, hA_2A_ and hA_3_ ARs while at the hA_2B_ AR the 1,3-dimethyl substitution seems to guarantee the best shape complementarity in the orthosteric binding pocket.

Secondly, all newly synthesized analogues were subjected to the same docking protocol. In particular, at the hA_3_ AR all compounds **4–7** showed an appreciable receptor complementarity even if two distinct plausible binding modes are detected (hereafter named A and B) analyzing all docking poses. In the binding mode A, the 1-benzyl-1*H*-pyrazole moiety is placed similarly to the CVT-like compounds previously described, with these specific interacting residues: Gln167 (EL2), Leu264 (7.35), Tyr265 (7.36). The substituent at the N5 position is inserted in an accessory pocket defined by Ser181 (5.42), Leu246 (6.51) and Ser247 (6.52). This pocket is located deeper in the TM bundle, under the Asn250 (6.51), and it is delimited by TM3 and TM5 and by Trp243 (6.48). Interestingly, this accessory pocket is probably the most relevant structural difference among the hypothetical binding sites of the AR subtypes. In particular, the Ser247 (6.52) in the hA_3_ AR is not conserved in the other subtypes, replaced by an histidine in the other subtypes. This mutation significantly perturbs the shape and the of the hA_3_ AR orthosteric binding pocket compared to those of all the other subtypes. More interestingly, this histidine has been reported to participate in both agonist and antagonist binding to the hA_2A_ AR by mutagenesis studies. [51,52] Also Ser181 (5.42) is a peculiarity of the hA_3_ AR being replaced by conserved asparagine in the other AR subtypes.

**Fig 6:**
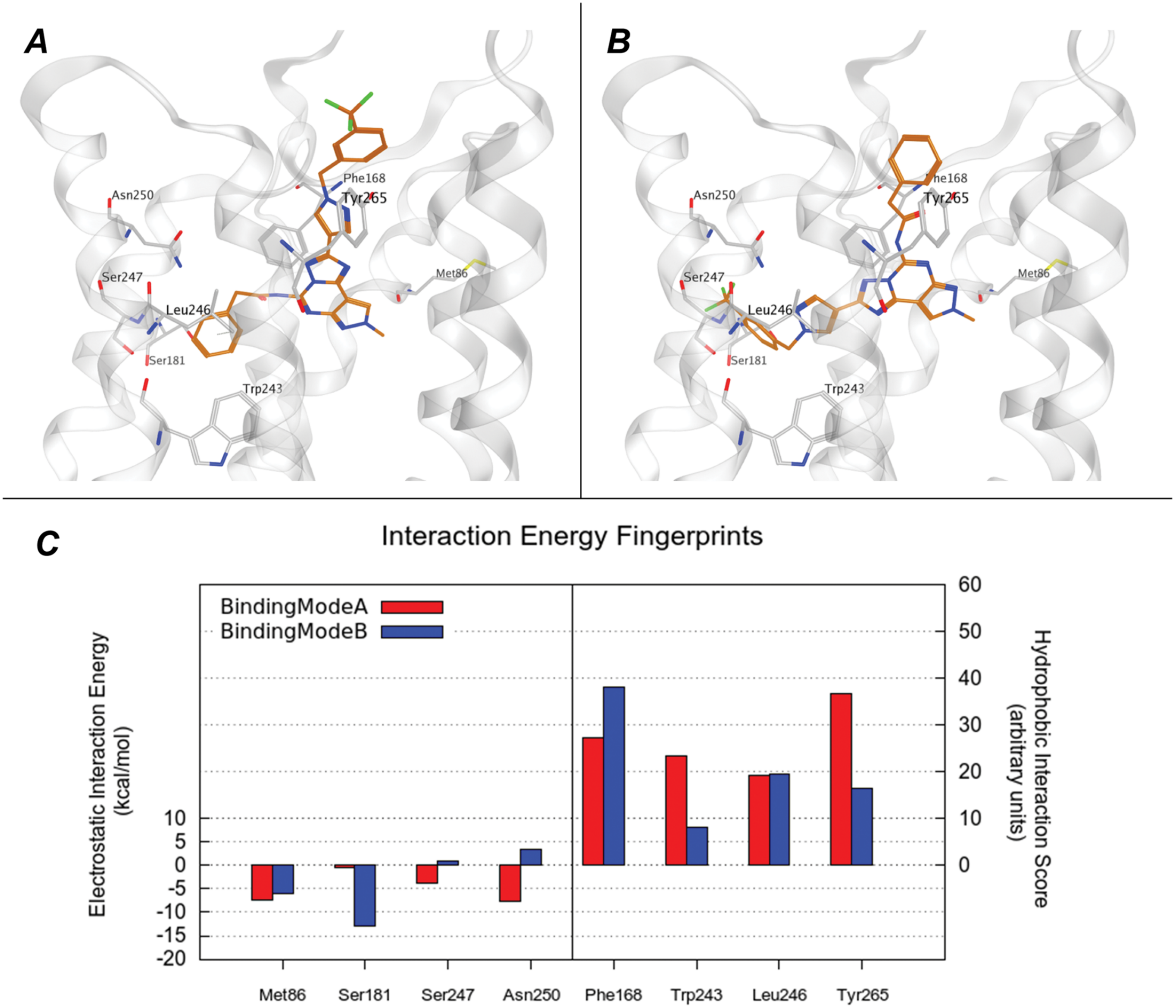
Binding mode of compound 6 at the hA_3_ AR. (A) (B) Hypothetical binding mode A and B of newly synthetized compounds to hA_3_ AR. The most potent derivative, **6**, was selected as example and is represented as orange stick. Subsets of hA_3_ AR residues, involved in the binding, are coloured in light grey. (C) The electrostatic and hydrophobic contributes to interaction energy calculated for the residue mostly involved in the binding are reported compound **6** in the conformation reported in panel A (in red) and B (in blue). Electrostatic energy values are expressed in kcal mol^–1^, whereas hydrophobic scores are expressed in arbitrary hydrophobic units.

However, in the hypothetical binding mode A the Asn250 (6.55) residue is not directly involved in the interaction with the compounds (**4**–**7**), although a water-mediated interaction could not be excluded. As an instance of binding mode A the pose of most potent analogue (**6**) is shown in Fig 6A, while the superposition of the receptor complex for **36** and **6** is reported in S4 Fig.

In the binding mode B, the position of the substituent at the C2 and N5 positions is inverted, while the orientations of the PTP core and of the methyl at N8 position are retained in the same region occupied in the binding mode A (Fig 6B and S4B Fig). The two distinct poses show similar interactions networks as demonstrated by comparing the Interaction Energy (IE) plots where the per-residue electrostatic and hydrophobic contributions are calculated (S4C Fig). Even a more quantitative comparison of the IE contributions for the most important residues reveals similar profile (Fig 6C). On the contrary, all analogues when docked into hA_1_, hA_2A_ and hA_2B_ ARs did not show a stabilized binding pose comparable to those observed for the hA_3_ AR. In particular compounds **4–7**, that are characterized by large substituents in both C2 and N5 positions, seem not to be able to occupy the accessory pocked that we have previously described characterizing the orthosteric binding site of the hA_3_ AR. Also in this case, the analysis of the Interaction Energy (IE) plots supports the hypothesis of the complementary lacking when compounds **4–7** interact with hA_1_, hA_2A_ and hA_2B_ ARs. Again, the most relevant difference can ascribed to the missing interaction with the residues delimiting the accessory cavity, as shown in S5 Fig.

The physicochemical and ADME (absorption, distribution, metabolism, and excretion) properties of the newly synthesized compounds (**3–7**) were calculated *in silico* and compared to those calculated for CVT-like derivatives and reference hA_3_ AR antagonists recently reviewed by Borea *et al*. (S1 Table). [53] The PTP scaffold in comparison to the xanthine core shows a deterioration of the pharmacokinetic (PK) profile. In particular, the partition coefficient cLogP (4.49 and 2.87 for compound **6** and CVT 6975 (**1**), respectively) and the solubility are affected. Nevertheless, compound **6** and CVT 6975 (**1**) present similar ADME properties. The limitations in the pharmacokinetic profile is a common issue for the selective hA_3_ AR antagonists already reported in literature. With only few exceptions, the gain in selectivity over the other human ARs subtypes is associated with a PK profile deterioration.

## Conclusions

We have presented a novel series of pyrazolo[4,3-*e*][1,2,4]triazolo[1,5-*c*]pyrimidines bearing a 1-(3-trifluoromethyl-benzyl)1*H*-pyrazol-4-yl moiety at the C2 position in order to explore the effect on affinity and selectivity profiles at the four ARs. In particular, the 1-(3-trifluoromethyl-benzyl)1*H*-pyrazol-4-yl group when attached at the 8 position of xanthine derivatives, such as CVT-6975 (**1**), is known to confer high affinity for the hA_2B_ AR. Curiously, the synthesized compounds were inactive at the hA_2B_ AR but demonstrated activity as potent and selective antagonist of the hA_3_ AR. A molecular docking study was performed in order to rationalize the influence of a 1-(3-trifluoromethyl-benzyl)-1*H*-pyrazole branch. We observed that only the hA_3_ AR has the topological features to accommodate this new series in agreement with experimental data. Interestingly, the ability to host such a big moiety can be ascribed to an accessory binding pocket present in the hA_3_ AR, which is formed by specific unconserved residues. Two possible binding modes were proposed in which the substituents in C2 and N5 are reciprocally inverted. Further studies will be needed to validate which one is predominant or if they may co-exist. Although derivatives more potent at the hA_3_ AR, and with a better drug-like properties than compound **6,** have been already reported in literature, our derivative shows high selectivity for hA_3_ AR over the other ARs subtypes (S1 Table), which is comparable to other selective antagonists already reported. [53] More interestingly, the high affinity obtained by introducing long chains, such as a benzyl-1*H*-pyrazole, at the C2 position, opens to new possibilities in the development of new derivatives bearing PTP or PTP-derived simplified scaffolds. In addition, in the binding mode A, the chain at the C2 position is directed to the solvent exposed area of the hA_3_ AR binding pocket, suggesting a new anchoring point for further derivatization (e.g. with fluorophore for detection purposes).

## Supporting Information

S1 Fig Competition of compounds 6 and 4 for A_3_ receptor binding. Both compounds show high affinity binding to hA_3_ ARs as shown by competition for the A_3_ selective agonist [^3^H]HEMADO. Representative curves (total binding) from single experiments with K_i_ values of 17 and 39 nM for compounds **6** and **4**, respectively, are reported.
(PDF)

S2 Fig Competition of compound 3 for A_1_ receptor binding. Compound **3** shows in a radioligand competition assay with the A_1_ selective radioligand [^3^H]CCPA a K_i_ value of 764 nM. The curve shows total binding to hA_1_ ARs from a representative single experiment.
(PDF)

S3 Fig Competition of compound 3 for A_2A_ receptor binding. Compound **3** shows in a radioligand competition assay with the nonselective radioligand [^3^H]NECA a K_i_ value of 6820 nM. The curve shows total binding to hA_2A_ ARs from a representative single experiment.
(PDF)

S4 Fig Hypothetical binding modes of compound 6 superimposed to compound 36 at the hA_3_ AR and electrostatic and hydrophobic contributions maps for compound 6. The hypothetical binding modes (A and B respectively indicated) of compound **6** are reported superposed to the coordinates of compound **36** to reveal the similarity in the accommodation of the common 1-(3-Trifluoromethyl-benzyl)-1H-pyrazole residue. The coordinates of compound **36** in B are obtained from a secondary docking solution. (C) Per residue electrostatic interaction energy map and per residue hydrophobic interaction score map. The maps are calculated for a selected pose of compound **6** inside the hA_3_ AR binding site. Electrostatic energy values are expressed in kcal mol–1, whereas hydrophobic scores are expressed in arbitrary hydrophobic units.
(TIF)

S5 Fig Comparison of the contribution to the docking score of the key residue for the binding of compound 6 to hA_3_ AR according molecular docking studies. The contributes to electrostatic and hydrophobic energy interactions for hA_1_, hA_2A_, hA_2B_ and hA_3_ ARs are reported in panels A, B, C and D respectively. In panel D, the profiles of the two predominant binding modes for hA_3_ AR, A (red) and B (blue), are showed. In Panel E the location of residues Met86, Ser181, Ser247 and Asn250 (in cyan) and Phe168, Trp243, Leu246 and Tyr265 (in green) in the A_3_ AR and the corresponding residues in the others AR subtypes is indicated by the ball representation of alpha Carbon atoms.
(TIF)

S1 Table Selectivity profile and predicted physicochemical and ADME properties of references and newly synthesized compounds (3–7). (DOCX)
