# Supplementary figures and images for: The Influence of the 1-(3-Trifluoromethyl-Benzyl)-1H-Pyrazole-4-yl Moiety on the Adenosine Receptors Affinity Profile of Pyrazolo[4,3-e][1,2,4]Triazolo[1,5-c]Pyrimidine Derivatives
Source: PLoS One. 2015 Dec 1;10(12):e0143504. doi: 10.1371/journal.pone.0143504 (PMC4666649; doi:10.1371/journal.pone.0143504)

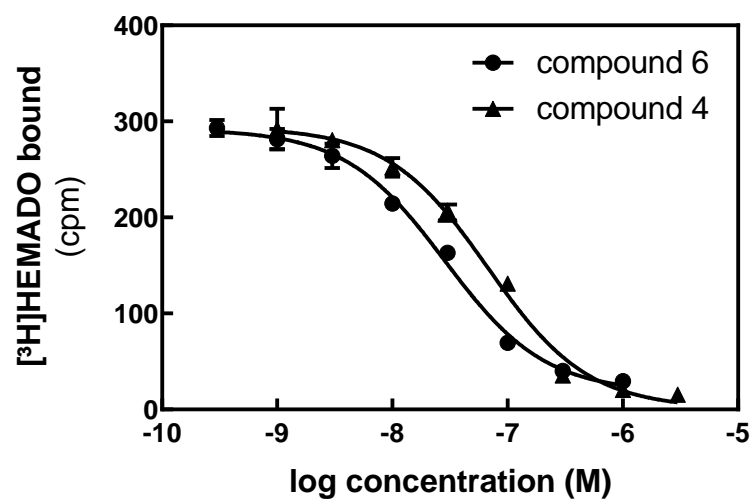

Supplement: S1 Fig — Both compounds show high affinity binding to hA3 ARs as shown by competition for the A3 selective agonist [3H]HEMADO. Representative curves (total binding) from single experiments with Ki values of 17 and 39 nM for compounds 6 and 4, respectively, are reported. (PDF) [file pone.0143504.s001.pdf]

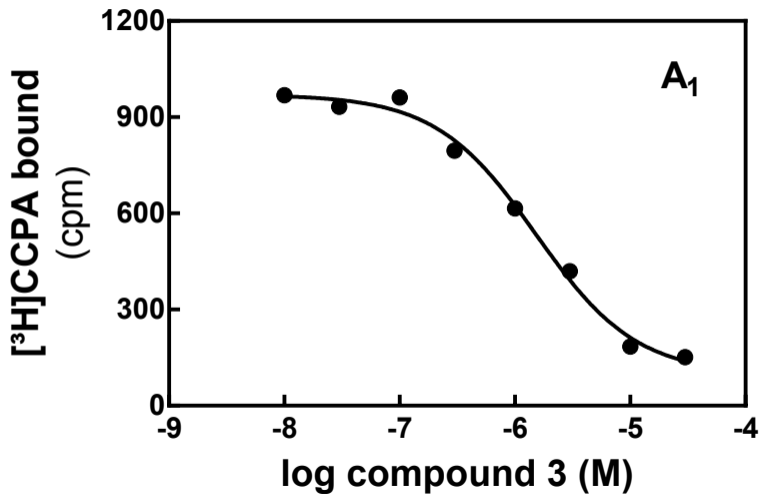

Supplement: S2 Fig — Compound 3 shows in a radioligand competition assay with the A1 selective radioligand [3H]CCPA a Ki value of 764 nM. The curve shows total binding to hA1 ARs from a representative single experiment. (PDF) [file pone.0143504.s002.pdf]

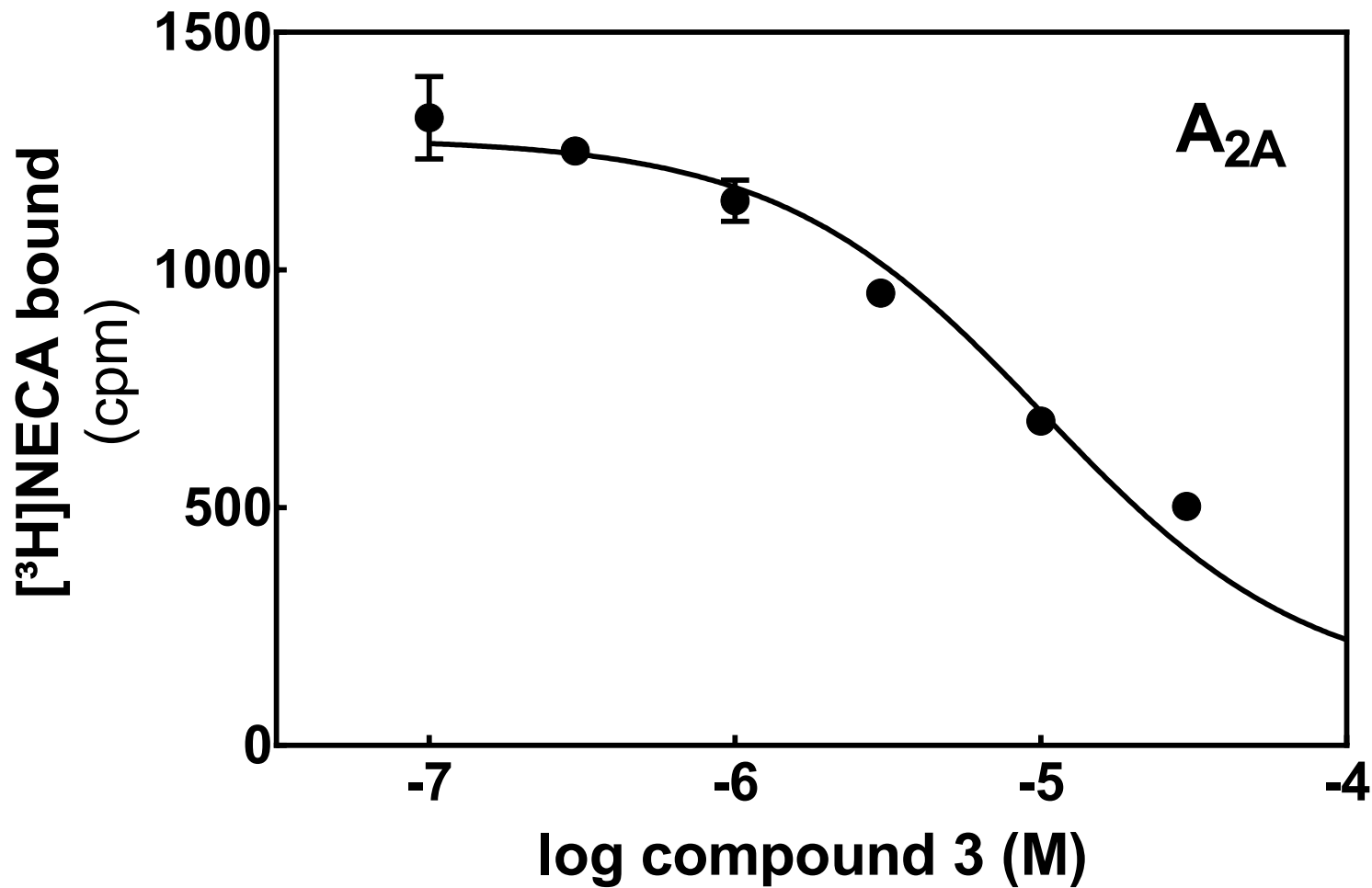

Supplement: S3 Fig — Compound 3 shows in a radioligand competition assay with the nonselective radioligand [3H]NECA a Ki value of 6820 nM. The curve shows total binding to hA2A ARs from a representative single experiment. (PDF) [file pone.0143504.s003.pdf]

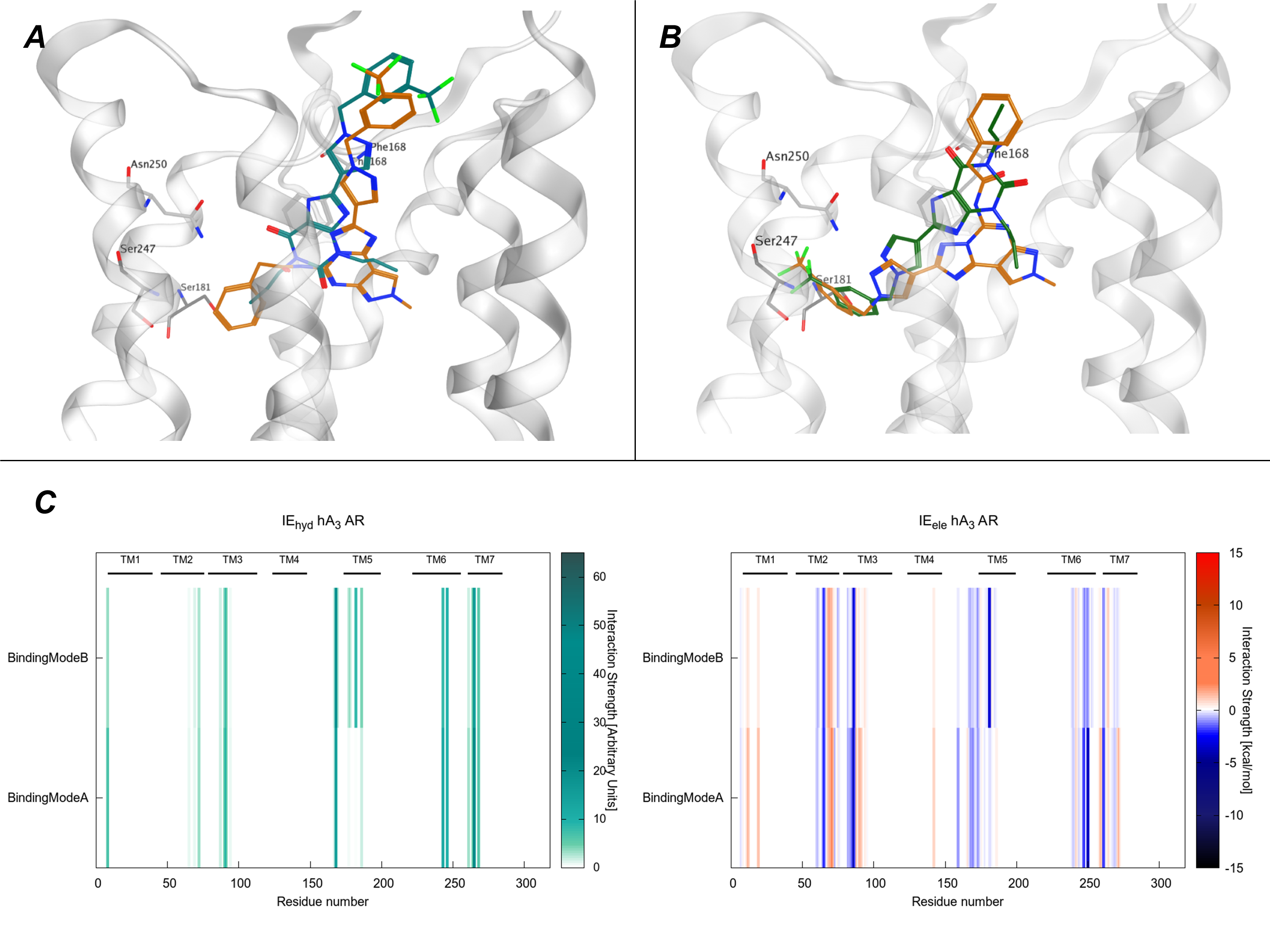

Supplement: S4 Fig — The hypothetical binding modes (A and B respectively indicated) of compound 6 are reported superposed to the coordinates of compound 36 to reveal the similarity in the accommodation of the common 1-(3-Trifluoromethyl-benzyl)-1H-pyrazole residue. The coordinates of compound 36 in B are obtained from a secondary docking solution. (C) Per residue electrostatic interaction energy map and per residue hydrophobic interaction score map. The maps are calculated for a selected pose of compound 6 inside the hA3 AR binding site. Electrostatic energy values are expressed in kcal mol–1, whereas hydrophobic scores are expressed in arbitrary hydrophobic units. (TIF) [file pone.0143504.s004.TIF]

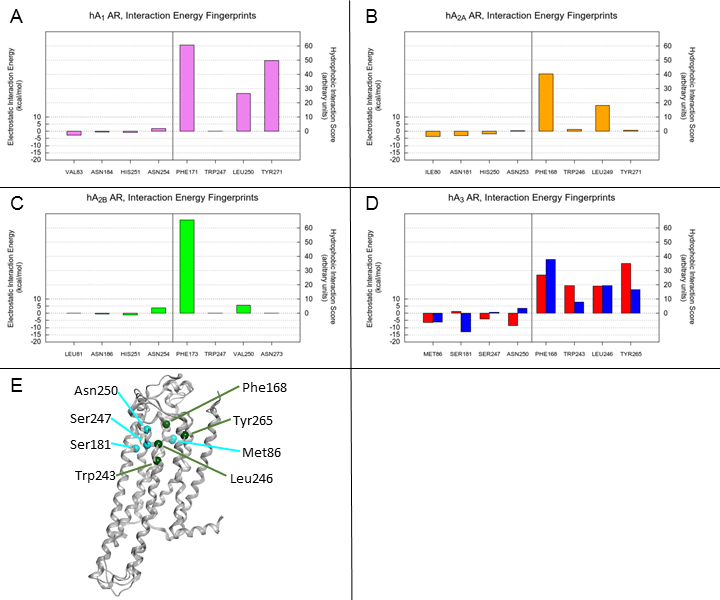

Supplement: S5 Fig — The contributes to electrostatic and hydrophobic energy interactions for hA1, hA2A, hA2B and hA3 ARs are reported in panels A, B, C and D respectively. In panel D, the profiles of the two predominant binding modes for hA3 AR, A (red) and B (blue), are showed. In Panel E the location of residues Met86, Ser181, Ser247 and Asn250 (in cyan) and Phe168, Trp243, Leu246 and Tyr265 (in green) in the A3 AR and the corresponding residues in the others AR subtypes is indicated by the ball representation of alpha Carbon atoms. (TIF) [file pone.0143504.s005.tif]
