# Supplementary material for: The Influence of the 1-(3-Trifluoromethyl-Benzyl)-1H-Pyrazole-4-yl Moiety on the Adenosine Receptors Affinity Profile of Pyrazolo[4,3-e][1,2,4]Triazolo[1,5-c]Pyrimidine Derivatives
Source: PLoS One. 2015 Dec 1;10(12):e0143504. doi: 10.1371/journal.pone.0143504 (PMC4666649; doi:10.1371/journal.pone.0143504)
Supplement: S1 Table — (DOCX) [file pone.0143504.s006.docx]

| **Table S1.** Selectivity profile and predicted physicochemical and ADME properties of references and newly synthesized compounds.   \| name \| logS^a^ \| logP^b^ \| 2C9 pKi^c^ \| hERG pIC_50_^d^ \| BBB log([brain]:[blood])^e^ \| HIA category^f^ \| P-gp category^g^ \| 2D6 affinity category^h^ \| PPB90 category^i^ \| HBD^l^ \| HBA^m^ \| TPSA^n^ \| Flexibility^o^ \| K_i_, hA_1_/hA_3_^p^ \| K_i_, hA_2a_/hA_3_^q^ \| K_i_, hA_2b_/hA_3_^r^ \| \| --- \| --- \| --- \| --- \| --- \| --- \| --- \| --- \| --- \| --- \| --- \| --- \| --- \| --- \| --- \| --- \| --- \| \| 3 \| 1.70 \| 3.22 \| 5.90 \| 5.64 \| -0.57 \| - \| yes \| high \| high \| 1 \| 9 \| 104.7 \| 0.12 \| 3∙10^-1^ \| 4 \| 4 \| \| 4 \| 0.65 \| 4.39 \| 6.42 \| 6.20 \| -0.77 \| - \| yes \| high \| high \| 2 \| 11 \| 119.8 \| 0.18 \| 83 \| 53 \| 214 \| \| 5 \| 0.77 \| 4.34 \| 6.15 \| 5.97 \| -0.49 \| - \| yes \| high \| high \| 1 \| 10 \| 107.8 \| 0.16 \| 185 \| 725 \| 72 \| \| 6 \| 0.73 \| 4.49 \| 6.34 \| 5.91 \| -0.76 \| - \| yes \| high \| high \| 1 \| 10 \| 107.8 \| 0.18 \| > 9091 \| >9091 \| >909 \| \| 33 \| 1.93 \| 3.90 \| 5.22 \| 5.51 \| -1.03 \| + \| yes \| low \| high \| 1 \| 8 \| 90.5 \| 0.21 \| 3 \| 4 \| 2.5∙10^-1^ \| \| 36 \| 1.71 \| 4.19 \| 5.47 \| 5.47 \| -0.95 \| + \| yes \| high \| high \| 1 \| 8 \| 90.5 \| 0.22 \| 1 \| 3 \| 9∙10^-2^ \| \| CVT 6975[2] \| 2.08 \| 2.87 \| 5.65 \| 4.95 \| -0.90 \| + \| yes \| high \| high \| 1 \| 8 \| 90.5 \| 0.13 \| 1 \| 1 \| 1∙10^-3^ \| \| MRE3005F20  (HCl)[3] \| 1.30 \| 3.24 \| 6.20 \| 5.80 \| -0.85 \| + \| yes \| medium \| high \| 2 \| 11 \| 124.4 \| 0.20 \| 35000 \| 10000 \| 25000 \| \| MRE3008F20 \| 1.34 \| 3.41 \| 6.21 \| 5.91 \| -0.84 \| + \| yes \| medium \| high \| 2 \| 11 \| 124.4 \| 0.22 \| 1463 \| 173 \| 2560 \| \| OT-7999 \| 1.78 \| 4.25 \| 5.87 \| 5.81 \| -0.63 \| + \| yes \| high \| high \| 1 \| 6 \| 71.8 \| 0.17 \| >10526 \| >10526 \| N.D. \| \| PSB-10 \| 0.26 \| 4.48 \| 5.81 \| 4.83 \| -0.21 \| + \| no \| high \| high \| 1 \| 5 \| 52.2 \| 0.07 \| 3864 \| 6136 \| N.D. \| \| PSB-11 \| 1.87 \| 2.57 \| 5.15 \| 4.78 \| -0.33 \| + \| no \| high \| low \| 1 \| 5 \| 52.2 \| 0.08 \| 701 \| 547 \| 897 \| \| KF-26777 \| 1.46 \| 3.01 \| 5.57 \| 5.13 \| -0.29 \| + \| no \| medium \| low \| 1 \| 5 \| 52.2 \| 0.11 \| 9000 \| 2350 \| 3100 \| \| MRS1191 \| -1.32 \| 5.96 \| 5.84 \| 5.56 \| -0.25 \| + \| Yes \| high \| high \| 1 \| 5 \| 68.12 \| 0.23 \| >318 \| >318 \| >318 \| \| MRS1220 \| 0.35 \| 4.58 \| 6.04 \| 5.44 \| -0.47 \| + \| Yes \| medium \| high \| 1 \| 7 \| 85.32 \| 0.15 \| 391 \| 42 \| N.D. \| \| MRS1523 \| -0.01 \| 5.37 \| 5.17 \| 5.33 \| 0.50 \| + \| yes \| high \| high \| 0 \| 4 \| 56.26 \| 0.38 \| >529 \| 194 \| >529 \| |
| --- | --- | --- | --- | --- | --- | --- | --- | --- | --- | --- | --- | --- | --- | --- | --- | --- | --- | --- | --- | --- | --- | --- | --- | --- | --- | --- | --- | --- | --- | --- | --- | --- | --- | --- | --- | --- | --- | --- | --- | --- | --- | --- | --- | --- | --- | --- | --- | --- | --- | --- | --- | --- | --- | --- | --- | --- | --- | --- | --- | --- | --- | --- | --- | --- | --- | --- | --- | --- | --- | --- | --- | --- | --- | --- | --- | --- | --- | --- | --- | --- | --- | --- | --- | --- | --- | --- | --- | --- | --- | --- | --- | --- | --- | --- | --- | --- | --- | --- | --- | --- | --- | --- | --- | --- | --- | --- | --- | --- | --- | --- | --- | --- | --- | --- | --- | --- | --- | --- | --- | --- | --- | --- | --- | --- | --- | --- | --- | --- | --- | --- | --- | --- | --- | --- | --- | --- | --- | --- | --- | --- | --- | --- | --- | --- | --- | --- | --- | --- | --- | --- | --- | --- | --- | --- | --- | --- | --- | --- | --- | --- | --- | --- | --- | --- | --- | --- | --- | --- | --- | --- | --- | --- | --- | --- | --- | --- | --- | --- | --- | --- | --- | --- | --- | --- | --- | --- | --- | --- | --- | --- | --- | --- | --- | --- | --- | --- | --- | --- | --- | --- | --- | --- | --- | --- | --- | --- | --- | --- | --- | --- | --- | --- | --- | --- | --- | --- | --- | --- | --- | --- | --- | --- | --- | --- | --- | --- | --- | --- | --- | --- | --- | --- | --- | --- | --- | --- | --- | --- | --- | --- | --- | --- | --- | --- | --- | --- | --- | --- | --- | --- | --- | --- | --- | --- | --- | --- | --- | --- | --- | --- | --- | --- | --- | --- | --- | --- | --- | --- | --- | --- | --- | --- | --- | --- | --- | --- | --- | --- | --- | --- | --- | --- | --- | --- | --- | --- | --- | --- | --- |

^a^aqueous solubility (logS, µM), preferably >1; ^b^logarithm of partition coefficient between *n*-octanol and water (*c*logP), preferably 0<clogP<3.6; ^c^CYP2C9 cytochrome metabolism (CYP2C9 affinity, µM), preferably ≤ 6; ^d^hERG channel inhibition (pIC_50_), preferably ≤ 5; ^e^blood brain barrier (BBB) penetration in terms of logarithm brain-blood partition; ^f^human intestinal absorption (HIA), (+) indicates absorption ≥ 30% and (-) indicates absorption < 30%; ^g^P-glycoprotein binding (P-gp), "yes" indicates a substrate and "no" indicates a non-substrate; ^h^CYP2D6 cytochrome metabolism (CYP2D6 affinity, µM), "low" indicates pKi < 5, "medium" indicates 5 < pKi < 6, "high" indicates 6 < pKi < 7, "very high" indicates pKi > 7; ^i^plasma protein binding (PPB90), "low" indicates < 90% of compound bound to plasma proteins, "high" indicates ≥ 90% of compound bound to plasma proteins; ^l^number of hydrogen bond donor (HBD); ^m^number of hydrogen bond acceptor (HBA); ^n^topological polar surface area (TPSA); ^o^flexibility; ^p-r^selectivity profile among ARs subtypes. The Affinity values were derived from Borea et al.[1] unless otherwise indicated. N.D., no data available. All properties were calculated using *StarDrop^TM^*, version 6.0 [4].

**References**

1 Borea PA, Varani K, Vincenzi F, Baraldi PG, Tabrizi MA, Merighi S, Gessi S (2015) The A3 adenosine receptor: history and perspectives. Pharmacol Rev 67:74-102

2 Kalla RV, Elzein E, Perry T, Li X, Palle V, Varkhedkar V, Gimbel A, Maa T, Zeng D, Zablocki J (2006) Novel 1,3-disubstituted 8-(1-benzyl-1H-pyrazol-4-yl) xanthines: high affinity and selective A2B adenosine receptor antagonists. J Med Chem 49:3682-3692

3 Maconi A, Pastorin G, Da Ros T, Spalluto G, Gao ZG, Jacobson KA, Baraldi PG, Cacciari B,Varani K, Moro S, Borea PA (2002) Synthesis, biological properties, and molecular modeling investigation of the first potent, selective, and water-soluble human A(3) adenosine receptor antagonist. J Med Chem 45:3579–3582

4 Stardrop^TM^, version 6.0; Optibrium Ltd: 7221 Cambridge Research Park, Beach Drive, Cambridge CB25 9TL, UK
